# Supplementary material for: Comorbidity and clinical factors associated with COVID-19 critical illness and mortality at a large public hospital in New York City in the early phase of the pandemic (March-April 2020)
Source: PLoS One. 2020 Nov 23;15(11):e0242760. doi: 10.1371/journal.pone.0242760 (PMC7682848; doi:10.1371/journal.pone.0242760)
Supplement: S1 Table — (DOCX) [file pone.0242760.s001.docx]

|  | **S1 Table. Socio-demographics, Medical History, Admission Laboratory Markers, Treatment, and Hospital Outcomes for Patients Not Requiring Supplemental Oxygen (n=65)** | |  |
| --- | --- | --- | --- |
|  |  | **No Supplemental Oxygen (n=65)** |  |
|  | **Sex, n(%)** |  |  |
|  | Male | 45 (69.2%) |  |
|  | Female | 20 (30.8%) |  |
|  | **Age, median (IQR)** | 55 (45 - 62) |  |
|  | **Age Category, n(%)** |  |  |
|  | <50 | 25 (38.5%) |  |
|  | 50-64 | 25 (38.5%) |  |
|  | 65+ | 15 (23.1%) |  |
|  | **Race/Ethnicity, n(%)** |  |  |
|  | A. Indian or Alaska N. | 0 (0%) |  |
|  | Asian | 3 (4.6%) |  |
|  | Black | 19 (29.2%) |  |
|  | Hispanic / Latinx | 4 (6.2%) |  |
|  | White | 5 (7.7%) |  |
|  | Other | 31 (47.7%) |  |
|  | Not Recorded or Unknown | 3 (4.6%) |  |
|  | **BMI, median (IQR), n=64** | **27.2 (22.6 - 32.0)** |  |
|  | **Medical Comorbidities, n(%)** |  |  |
|  | Cardiovascular Comorbidity^1^ | 36 (55.4%) |  |
|  | Pulmonary Comorbidity^2^ | 7 (10.8%) |  |
|  | Renal Comorbidity^3^ | 15 (23.1%) |  |
|  | CKD | 8 (12.3%) |  |
|  | ESRD | 7 (10.8%) |  |
|  | Type 2 Diabetes | 20 (30.8%) |  |
|  | Immunosuppression | 3 (4.6%) |  |
|  | HIV | 1 (1.5%) |  |
|  | Cirrhosis | 3 (4.6%) |  |
|  | Malignancy | 3 (4.6%) |  |
|  | Dementia | 1 (1.5%) |  |
|  | Obesity (BMI >30.0), n=64 | 21 (32.8%) |  |
|  | No Listed Comorbidities^4^ | 17 (26.2%) |  |
|  | **Pregnancy, n(%)** | 0 (0%) |  |
|  | **Homelessness, n(%)** | 18 (27.7%) |  |
|  | **Symptoms** |  |  |
|  | Fever | 40 (61.5%) |  |
|  | Cough | 37 (56.9%) |  |
|  | Dyspnea | 18 (27.7%) |  |
|  | Chest Pain | 13 (20.0%) |  |
|  | Diarrhea | 14 (21.5%) |  |
|  | Myalgias | 18 (27.7%) |  |
|  | Anosmia | 1 (1.5%) |  |
|  | Altered Mental Status | 7 (10.8%) |  |
|  | Headache | 10 (15.4%) |  |
|  | Syncope | 5 (7.7%) |  |
|  | **Symptom Duration, median (IQR), n=49** | 3 (1 - 6) |  |
|  | **NEWS score, median (IQR)** | 2 (1 - 3) |  |
|  | **NEWS Score Category, n(%)** |  |  |
|  | Low Risk (0-4) | 55 (84.6%) |  |
|  | Medium Risk (5-6) | 4 (6.2%) |  |
|  | High Risk (7+) | 6 (9.2%) |  |
|  | **Admission Lab Tests, median (IQR)** |  |  |
|  | WBC (10^3^/μl) | 5.8 (4.5 - 8.0) |  |
|  | ANC (10^3^/μl) | 4.0 (3.1 - 5.6) |  |
|  | ALC (10^3^/μl) | 1.0 (0.62 - 1.4) |  |
|  | CRP (mg/L), n=39 | 49.5 (8.10 - 82.9) |  |
|  | D-dimer (ng/mL), n=31 | 282 (177 - 802) |  |
|  | Ferritin (ng/mL), n=35 | 351 (162 - 850) |  |
|  | LDH (IU/mL), n=41 | 292 (250 - 432) |  |
|  | **Admission Lab Tests, n(%)** |  |  |
|  | Lactate >2.0 (mmol/L), n=31 | 4 (12.9%) |  |
|  | Troponin >= 0.05 (ng/mL), n=34 | 4 (11.8%) |  |
|  | AST >40 (U/L), n=59 | 25 (42.4%) |  |
|  | ALT >36 (U/L), n=59 | 19 (32.2%) |  |
|  | **Smoking^5^, n(%)** |  |  |
|  | Current Smoker | 6 (9.2%) |  |
|  | Former Smoker | 15 (23.1%) |  |
|  | Never Smoker | 29 (44.6%) |  |
|  | Unknown / Not Recorded | 15 (23.1%) |  |
|  | **Exposures, n(%)** |  |  |
|  | Known COVID+ Contact | 4 (6.2%) |  |
|  | Sick Household Contact | 8 (12.3%) |  |
|  | International Travel | 0 (0%) |  |
|  | Healthcare Worker | 3 (4.6%) |  |
|  | No Recorded Exposure | 50 (76.9%) |  |
|  | **Treatment, n(%)** |  |  |
|  | Lopinavir/Ritonavir | 3 (4.6%) |  |
|  | Hydroxychloroquine (HCQ) | 9 (13.8%) |  |
|  | HCQ + Azithromycin | 13 (20.0%) |  |
|  | Any HCQ | 22 (33.8%) |  |
|  | Tocilizumab | 0 (0%) |  |
|  | Remdesivir Study Enrollment | 0 (0%) |  |
|  | Antimicrobials | 25 (38.5%) |  |
|  | Steroids | 1 (1.5%) |  |
|  | None of the Above | 24 (36.9%) |  |
|  | **Renal Outcomes, n(%)** |  |  |
|  | Baseline ESRD | 7 (10.8%) |  |
|  | No ESRD | 58 (89.2%) |  |
|  | No AKI^6^ | 49 (84.5%) |  |
|  | AKI w/o RRT^6^ | 8 (13.8%) |  |
|  | AKI w/ RRT^6^ | 1 (1.7%) |  |
|  | **Hospital Outcomes, n(%)** |  |  |
|  | Mortality | 3 (4.6%) |  |
|  | Remain in Hospital by HD 30 | 7 (10.8%) |  |
|  | Discharged by HD 30 | 55 (84.6%) |  |
|  | All variables were calculated for the full sample of 65 patients unless different (n) indicated in variable column. Median and interquartile range (IQR) presented for continuous variables. Count and proportion presented for remaining categorical variables.  ^1^Hypertension, Heart Failure, Stroke or Transient Ischemic Attack, | |  |
|  | Coronary Artery Disease |  |  |
|  | ^2^Asthma, Chronic Obstructive Pulmonary Disease (COPD), | |  |
|  | Obstructive Sleep Apnea, Interstitial Lung Disease | |  |
|  | ^3^Chronic Kidney Disease (CKD) or End-stage Renal Disease (ESRD) | | |
|  | ^4^Patients lacking all comorbid medical conditions included in this table.  ^5^No current or prior vaporizer usage was reported in this cohort.  ^6^Percentages reported from denominator of patients without baseline ESRD. | |  |
|  | Abbreviations: AKI Acute Kidney Injury; RRT Renal Replacement Therapy;  HD Hospital Day | | |
|  |  | |  |
|  |  |  |  |

|  |  |  |  |  |  |
| --- | --- | --- | --- | --- | --- |
|  |  |  |  |  |  |
|  |  |  |  |  |  |
