# Supplementary material for: Comorbidity and clinical factors associated with COVID-19 critical illness and mortality at a large public hospital in New York City in the early phase of the pandemic (March-April 2020)
Source: PLoS One. 2020 Nov 23;15(11):e0242760. doi: 10.1371/journal.pone.0242760 (PMC7682848; doi:10.1371/journal.pone.0242760)
Supplement: S2 Table — (DOCX) [file pone.0242760.s002.docx]

|  | **S2 Table. Smoking, Exposures, and Presenting Vital Signs for Patients with COVID-19 Illness Requiring Supplemental Oxygen (n=270)** | | | |  |
| --- | --- | --- | --- | --- | --- |
|  |  | **Never ICU (135)** | **ICU (135)** | **Total (270)** |  |
|  | **Smoking^1^, n(%)** |  |  |  |  |
|  | Current Smoker | 8 (5.9%) | 5 (3.7%) | 13 (4.8%) |  |
|  | Former Smoker | 22 (16.3%) | 18 (13.3%) | 40 (14.8%) |  |
|  | Never Smoker | 89 (65.9%) | 84 (62.2%) | 173 (64.1%) |  |
|  | Unknown / Not Recorded | 16 (11.9%) | 28 (20.7%) | 44 (16.3%) |  |
|  | **Exposures, n(%)** |  |  |  |  |
|  | Known COVID+ Contact | 12 (8.9%) | 9 (6.7%) | 21 (7.8%) |  |
|  | Sick Household Contact | 28 (20.7%) | 24 (17.8%) | 52 (19.3%) |  |
|  | International Travel | 4 (3.0%) | 1 (0.7%) | 5 (1.9%) |  |
|  | Healthcare Worker | 5 (3.7%) | 2 (1.5%) | 7 (2.6%) |  |
|  | No Recorded Exposure | 91 (67.4%) | 100 (74.1%) | 191 (70.7%) |  |
|  | **Abnormal Vital Signs (First 24 Hours of Admission), n(%)** |  |  |  |  |
|  | Fever | 78 (57.8%) | 79 (58.5%) | 157 (58.1%) |  |
|  | Tachycardia | 80 (59.3%) | 85 (63.0%) | 165 (61.1%) |  |
|  | Tachypnea | 83 (61.5%) | 95 (70.4%) | 178 (65.9%) |  |
|  | Hypotension | 5 (3.7%) | 22 (16.3%) | 27 (10.0%) |  |
|  | SpO2 <=93% | 95 (70.4%) | 106 (78.5%) | 201 (74.4%) |  |
|  | Data are reported as count and proportion for categorical variables.  ^1^No current or prior vaporizer usage was reported in this cohort. | | |  |  |
