# Supplementary material for: Comorbidity and clinical factors associated with COVID-19 critical illness and mortality at a large public hospital in New York City in the early phase of the pandemic (March-April 2020)
Source: PLoS One. 2020 Nov 23;15(11):e0242760. doi: 10.1371/journal.pone.0242760 (PMC7682848; doi:10.1371/journal.pone.0242760)
Supplement: Attachment — Submitted filename: PONE-D-20-23829_reviewer comments.pdf [file pone.0242760.s004.pdf]

PLOS ONE

Comorbidity and Clinical Factors Associated with COVID-19 Critical Illness and Mortality at a Large Public Hospital in New York City in the Early Phase of the Pandemic (March-April 2020)

--Manuscript Draft--

**Manuscript Number:**	PONE-D-20-23829
**Article Type:**	Research Article
**Full Title:**	Comorbidity and Clinical Factors Associated with COVID-19 Critical Illness and Mortality at a Large Public Hospital in New York City in the Early Phase of the Pandemic (March-April 2020)
**Short Title:**	COVID-19 at a Public Hospital in NYC
**Corresponding Author:**	Thomas Daniel Filardo, M.D.<br>NYU Langone Health<br>New York, NY UNITED STATES
**Keywords:**	COVID-19<br>Infectious Diseases<br>Hospital-Based Practice<br>Public Hospital
**Abstract:**	Background: Despite evidence of sociodemographic disparities in outcomes of COVID-19, little is known about characteristics and clinical outcomes of patients admitted to public hospitals during the COVID-19 outbreak.<br>Objective: To assess demographics, comorbid conditions, and clinical factors associated with critical illness and mortality among patients diagnosed with COVID-19 at a public hospital in New York City (NYC) during the first month of the COVID-19 outbreak.<br>Design: Retrospective chart review of patients diagnosed with COVID-19 admitted to NYC Health + Hospitals / Bellevue Hospital from March 9th to April 8th, 2020.<br>Results: A total of 337 patients were diagnosed with COVID-19 during the study period. Primary analyses were conducted among those requiring supplemental oxygen (272); half of these patients (136) were admitted to the intensive care unit (ICU). A majority were male (67.6%) and the median age was 58. Approximately a third (33.1%) of hypoxic patients managed outside the ICU required non-rebreather or non-invasive ventilation. Requirement of renal replacement therapy occurred in 42.0% of ICU patients. Overall, 30-day mortality among hypoxic patients was 28.7% (52.9% in the ICU, 4.4% outside the ICU). In adjusted analyses, risk factors associated with mortality included dementia (adjusted risk ratio (aRR) 2.11 95%CI 1.50-2.97), age 65 or older (aRR 2.0, 95%CI 1.33-3.0), obesity (aRR 1.36, 95%CI 1.07-1.74), and male sex (aRR 1.32, 95%CI 1.03-1.68).<br>Conclusion: COVID-19 demonstrated severe morbidity and mortality in critically ill patients. Modifications in care delivery outside the ICU allowed the hospital to effectively care for a surge of critically ill and severely hypoxic patients.
**Order of Authors:**	Thomas Daniel Filardo, M.D.
	Maria R Khan
	Noa Krawczyk
	Hayley Galitzer
	Savannah Karmen-Tuohy
	Megan Coffee
	Verity E. Schaye
	Benjamin Eckhardt
	Gabriel Cohen
**Additional Information:**	

Powered by Editorial Manager® and ProduXion Manager® from Aries Systems Corporation

Question	Response
**Financial Disclosure**<br><br>Enter a financial disclosure statement that describes the sources of funding for the work included in this submission. Review the submission guidelines for detailed requirements. View published research articles from *PLOS ONE* for specific examples.<br><br>This statement is required for submission and **will appear in the published article** if the submission is accepted. Please make sure it is accurate.<br><br>**Unfunded studies**<br>Enter: *The author(s) received no specific funding for this work.*<br><br>**Funded studies**<br>Enter a statement with the following details:<br>• Initials of the authors who received each award<br>• Grant numbers awarded to each author<br>• The full name of each funder<br>• URL of each funder website<br>• Did the sponsors or funders play any role in the study design, data collection and analysis, decision to publish, or preparation of the manuscript?<br>• **NO** - Include this sentence at the end of your statement: *The funders had no role in study design, data collection and analysis, decision to publish, or preparation of the manuscript.*<br>• **YES** - Specify the role(s) played.<br><br>* typeset	The authors received no specific funding for this work.
**Competing Interests**<br><br>Use the instructions below to enter a competing interest statement for this submission. On behalf of all authors, disclose any competing interests that could be perceived to bias this work—acknowledging all financial support and any other relevant financial or non-financial competing interests.<br><br>This statement **will appear in the published article** if the submission is	The authors have declared that no competing interests exist.

accepted. Please make sure it is accurate. View published research articles from *PLOS ONE* for specific examples.<br><br>**NO authors have competing interests**<br><br>Enter: *The authors have declared that no competing interests exist.*<br><br>**Authors with competing interests**<br><br>Enter competing interest details beginning with this statement:<br><br>*I have read the journal's policy and the authors of this manuscript have the following competing interests: [insert competing interests here]*<br><br>* typeset	
**Ethics Statement**<br><br>Enter an ethics statement for this submission. This statement is required if the study involved:<br><br>• Human participants<br>• Human specimens or tissue<br>• Vertebrate animals or cephalopods<br>• Vertebrate embryos or tissues<br>• Field research<br><br>Write "N/A" if the submission does not require an ethics statement.<br><br>General guidance is provided below. Consult the submission guidelines for detailed instructions. **Make sure that all information entered here is included in the Methods section of the manuscript.**	This study was approved by the Institutional Review Boards of New York University Grossman School of Medicine and New York City Health + Hospitals. Informed consent was not obtained due to minimal risk to enrolled participants; a waiver of informed consent was obtained from both Institutional Review Boards.

**Format for specific study types**<br><br>**Human Subject Research (involving human participants and/or tissue)**<br>• Give the name of the institutional review board or ethics committee that approved the study<br>• Include the approval number and/or a statement indicating approval of this research<br>• Indicate the form of consent obtained (written/oral) or the reason that consent was not obtained (e.g. the data were analyzed anonymously)<br><br>**Animal Research (involving vertebrate animals, embryos or tissues)**<br>• Provide the name of the Institutional Animal Care and Use Committee (IACUC) or other relevant ethics board that reviewed the study protocol, and indicate whether they approved this research or granted a formal waiver of ethical approval<br>• Include an approval number if one was obtained<br>• If the study involved *non-human primates*, add *additional details* about animal welfare and steps taken to ameliorate suffering<br>• If anesthesia, euthanasia, or any kind of animal sacrifice is part of the study, include briefly which substances and/or methods were applied<br><br>**Field Research**<br><br>Include the following details if this study involves the collection of plant, animal, or other materials from a natural setting:<br>• Field permit number<br>• Name of the institution or relevant body that granted permission	
**Data Availability**<br><br>Authors are required to make all data underlying the findings described fully available, without restriction, and from the time of publication. PLOS allows rare exceptions to address legal and ethical concerns. See the PLOS Data Policy and FAQ for detailed information.	Yes - all data are fully available without restriction

A Data Availability Statement describing where the data can be found is required at submission. Your answers to this question constitute the Data Availability Statement and **will be published in the article**, if accepted.<br><br>**Important:** Stating 'data available on request from the author' is not sufficient. If your data are only available upon request, select 'No' for the first question and explain your exceptional situation in the text box.<br><br>Do the authors confirm that all data underlying the findings described in their manuscript are fully available without restriction?	
**Describe where the data may be found in full sentences. If you are copying our sample text, replace any instances of XXX with the appropriate details.**<br><br>• If the data are **held or will be held in a public repository**, include URLs, accession numbers or DOIs. If this information will only be available after acceptance, indicate this by ticking the box below. For example: *All XXX files are available from the XXX database (accession number(s) XXX, XXX.).*<br>• If the data are all contained **within the manuscript and/or Supporting Information files**, enter the following: *All relevant data are within the manuscript and its Supporting Information files.*<br>• If neither of these applies but you are able to provide **details of access elsewhere**, with or without limitations, please do so. For example:<br><br>*Data cannot be shared publicly because of [XXX]. Data are available from the XXX Institutional Data Access / Ethics Committee (contact via XXX) for researchers who meet the criteria for access to confidential data.*<br><br>*The data underlying the results presented in the study are available from (include the name of the third party*	All relevant data are within the manuscript and its Supporting Information files.

and contact information or URL).<br>• This text is appropriate if the data are owned by a third party and authors do not have permission to share the data.<br><br>* typeset	
Additional data availability information:	

Powered by Editorial Manager® and ProduXion Manager® from Aries Systems Corporation

Manuscript

Click here to access/download;Manuscript;BHC Manuscript PLOS 7.30.20.docx

Comorbidity and Clinical Factors Associated with COVID-19 Critical Illness and Mortality at a Large Public Hospital in New York City in the Early Phase of the Pandemic (March-April 2020)

Short Title: COVID-19 at a Public Hospital in NYC

Thomas D. Filardo[1], Maria R. Khan[2], Noa Krawczyk[2], Hayley Galitzer[3], Savannah Karmen-Tuohy[3], Megan Coffee[1,4], Verity E. Schaye[4,5], Benjamin J. Eckhardt[1,4], Gabriel M. Cohen[1,4]

1: New York University Grossman School of Medicine, Division of Infectious Diseases and Immunology, New York, NY
2: New York University Grossman School of Medicine, Department of Population Health, New York, NY
3: New York University Grossman School of Medicine, New York, NY
4: NYC Health + Hospitals / Bellevue Hospital Center, New York, NY
5: New York University Grossman School of Medicine, Department of Internal Medicine, New York, NY

Corresponding Author:
Thomas D. Filardo, MD
718-316-1697
Thomas.Filardo@nyulangone.org

Abstract:

Background: Despite evidence of sociodemographic disparities in outcomes of COVID-19, little is known about characteristics and clinical outcomes of patients admitted to public hospitals during the COVID-19 outbreak.

Objective: To assess demographics, comorbid conditions, and clinical factors associated with critical illness and mortality among patients diagnosed with COVID-19 at a public hospital in New York City (NYC) during the first month of the COVID-19 outbreak.

Design: Retrospective chart review of patients diagnosed with COVID-19 admitted to NYC Health + Hospitals / Bellevue Hospital from March 9th to April 8th, 2020.

Results: A total of 337 patients were diagnosed with COVID-19 during the study period. Primary analyses were conducted among those requiring supplemental oxygen (272); half of these patients (136) were admitted to the intensive care unit (ICU). A majority were male (67.6%) and the median age was 58. Approximately a third (33.1%) of hypoxic patients managed outside the ICU required non-rebreather or non-invasive ventilation. Requirement of renal replacement therapy occurred in 42.0% of ICU patients. Overall, 30-day mortality among hypoxic patients was 28.7% (52.9% in the ICU, 4.4% outside the ICU). In adjusted analyses, risk factors associated with mortality included dementia (adjusted risk ratio (aRR) 2.11 95%CI 1.50-2.97), age 65 or older (aRR 2.0, 95%CI 1.33-3.0), obesity (aRR 1.36, 95%CI 1.07-1.74), and male sex (aRR 1.32, 95%CI 1.03-1.68).

Conclusion: COVID-19 demonstrated severe morbidity and mortality in critically ill patients. Modifications in care delivery outside the ICU allowed the hospital to effectively care for a surge of critically ill and severely hypoxic patients.

Introduction:

COVID-19, the disease caused by infection with severe acute respiratory syndrome coronavirus 2 (SARS-CoV-2) was first identified in China in late 2019 and subsequently spread globally. The WHO declared the outbreak of COVID-19 a pandemic on March 11, 2020. [1] In March of 2020, New York City (NYC) became the epicenter of the outbreak in the United States, experiencing over 219,030 cases, 18,800 confirmed deaths, and 4,624 probable deaths as of July 21, 2020. [2,3] During the peak of the COVID-19 surge, NYC hospitals were forced to adapt in order to meet the needs of an overwhelming number of cases; bed capacity was expanded and clinical triage and treatment algorithms were rapidly implemented.

COVID-19 presents with a broad spectrum of illness, ranging from asymptomatic or mild infections to severe disease leading to critical illness and multi-organ failure. Advanced age and comorbid medical conditions, such as hypertension and diabetes, have been shown to predispose individuals to severe illness due to COVID-19. [4-8] Prior studies have shown that patients admitted to the intensive care unit (ICU) due to COVID-19 have high mortality. [9-11] Furthermore, critical illness due to COVID-19 can be prolonged, straining hospital systems beyond their typical capacity. [10-12]

In this study we describe patients admitted to NYC Health + Hospitals / Bellevue Hospital Center (BHC), a large, academic, public safety-net hospital in NYC, with COVID-19 in the month following the hospital's first diagnosed case. In particular, we focus on comorbidities, clinical characteristics, and outcomes for patients admitted to ICU and non-ICU settings.

Methods:

Study Setting:

BHC is an 844-bed quaternary-care referral center for the NYC Health + Hospitals (NYCH+H) system. NYCH+H comprises 11 acute care hospitals, and is the public safety-net healthcare system for NYC. NYCH+H facilities were on the front lines of the response to the COVID-19 pandemic, serving vulnerable populations throughout the city. [13] This study was approved by the New York University Grossman School of Medicine and NYCH+H Institutional Review Boards.

Data

This is a retrospective review of the electronic medical record (EMR) of patients aged 18 and older admitted to BHC with laboratory-confirmed COVID-19 between March 9th 2020 and April 8th 2020. Laboratory-confirmed COVID-19 was defined as having a positive reverse transcriptase polymerase chain reaction (RT-PCR) test for SARS-CoV-2 virus. Patients were tested using a variety of diagnostic tests, including those performed by the NYC Department of Health and Mental Hygiene (DOHMH) (started 2/5/2020), as well as Labcorp (started 3/8/2020) and Bioreference (started 3/19/2020) commercial laboratories. BHC began using an in-house test (Cepheid GeneXpert Xpress) on 4/1/2020. Patients diagnosed at affiliated NYCH+H hospitals and transferred to BHC during the study period were included in this analysis, with data available via an integrated EMR. Incarcerated individuals were excluded as a vulnerable population.

Retrospective manual data extraction was conducted by a team of students using a standardized data capture tool. Data extracted from the EMR were entered into a secure REDCap (research electronic data capture) database. [14]

Measures

Data included demographics, comorbid conditions, presenting symptoms, admission vital signs and laboratory values, level of respiratory support, treatment, complications, and hospital course (i.e. ICU transfers, discharge, death). Laboratory values on hospital days 0, 1, and 2 were included as admission labs. Only the first available lab value was included if multiple were taken during this time-frame. If a patient was diagnosed with COVID-19 7 or more days after the date of hospital admission, the date of COVID-19 testing was considered hospital day 0 for this analysis. Otherwise, the day of admission was considered hospital day 0. Acute kidney injury (AKI) was defined as an increase in serum creatinine 0.3mg/dL above baseline in 48 hours or less. Use of therapies targeting SARS-CoV-2 and acute respiratory distress syndrome (ARDS) were gathered.

Our primary outcome was mortality at 30 days. Secondary outcomes included discharge from the hospital by 30 days, persistent hospitalization at 30 days, use of mechanical ventilation and other respiratory support, and incidence of AKI with or without requirement of renal replacement therapy

(RRT). Patients with baseline end-stage renal disease (ESRD) were excluded from outcomes regarding renal injury.

Patients were assessed by their highest level of oxygen and ventilatory support, in ascending order of supplementation: no supplemental oxygen, oxygen via nasal cannula or simple facemask at 6L/min or less, oxygen via non-rebreather (NRB), respiratory support via non-invasive ventilation or high-flow nasal cannula (NIV/HFNC), or mechanical ventilation.

The National Early Warning Score (NEWS) was calculated for patients based on admission vitals (temperature, heart rate, respiratory rate, systolic blood pressure, pulse oximetry) and altered mental status on presentation was assessed based on provider documentation. [15]

Data Analysis

We used bivariable analyses to calculate frequencies and/or means of patient background factors, symptoms, and comorbidities, clinical characteristics, and hospital outcomes by disease severity. We estimated binomial regression models for the associations between sociodemographics, comorbid medical conditions, and laboratory abnormalities with mortality. We evaluated whether laboratory values were more strongly associated with mortality according to sex, age, and comorbidity burden, testing effect modification by sex, age and comorbidity at the 0.15 level. Data analysis was completed in Stata, version 15.0 (Stata Corp., College Station, TX).

Results:

Study Population and Demographics:

Between March 9th and April 8th, 2020, a total of 337 adult patients eligible for inclusion in this study were diagnosed with COVID-19 at BHC. This cohort was split first by need for supplemental oxygen, with 65 patients (19.3%) never requiring supplemental oxygen. This group represented a heterogeneous population, including many admitted from congregate settings early in the outbreak for social isolation and individuals already hospitalized for other reasons (e.g. inpatient psychiatric care, acute inpatient rehab). Details regarding the 65 patients never requiring supplemental oxygen are available in Appendix Table 1. Primary analyses were conducted among those who required supplemental oxygen (N=272).

The remaining 272 patients requiring supplemental oxygen were divided by requirement of ICU admission, with 136 (50.0%) requiring ICU admission during the study period. The median age was 58, with 63 (23.2%) patients under age 50. Approximately two-thirds (67.6%) were male. Detailed information regarding race and ethnicity was available for 127 (46.7%) participants, with a minority identifying as white (9.2%). A total of 16 patients (5.9%) were homeless, either street- or shelter-domiciled (Table 1).

Table 1: Sociodemographics, Medical History, and Admission Laboratory Markers for Patients with COVID-19 Illness Requiring Supplemental Oxygen

	Supplemental Oxygen, Never ICU (136)	Supplemental Oxygen, ICU (136)	Total (272)	P-value[1]
**Sex**				

Male	88 (64.7%)	96 (70.6%)	184 (67.6%)	0.30
Female	48 (35.3%)	40 (29.4%)	88 (32.4%)	-
**Age**				
Median Age (IQR)	57 (48 - 67)	60 (52 - 68)	58 (50 - 67)	0.32
<50	37 (27.2%)	26 (19.1%)	63 (23.2%)	0.27
50-64	60 (44.1%)	64 (47.1%)	124 (45.6%)	-
65+	39 (28.7%)	46 (33.8%)	85 (31.2%)	-
**Race/Ethnicity**				
A. Indian or Alaska N.	0 (0%)	1 (0.7%)	1 (0.4%)	0.49
Asian	9 (6.6%)	12 (8.8%)	21 (7.7%)	-
Black	29 (21.3%)	27 (19.9%)	56 (20.6%)	-
Hispanic / Latinx	9 (6.6%)	15 (11.0%)	24 (8.8%)	-
White	16 (11.8%)	9 (6.6%)	25 (9.2%)	-
Other	62 (45.6%)	64 (47.1%)	126 (46.3%)	-
Not Recorded or Unknown	11 (8.1%)	8 (5.9%)	19 (7.0%)	-
**Medical / Social History**				
Cardiovascular Comorbidity[2]	66 (48.5%)	74 (54.4%)	140 (51.5%)	0.33
Pulmonary Comorbidity[3]	21 (15.4%)	15 (11.0%)	36 (13.2%)	0.28
Renal Comorbidity[4]	12 (8.8%)	20 (14.7%)	32 (11.8%)	0.13
CKD	4 (2.9%)	15 (11.0%)	19 (7.0%)	<0.01
ESRD	8 (5.9%)	5 (3.7%)	13 (4.8%)	0.39
Type 2 Diabetes	42 (30.9%)	48 (35.3%)	90 (33.1%)	0.44
Immunosuppression	6 (4.4%)	5 (3.7%)	11 (4.0%)	0.76
HIV	3 (2.2%)	2 (1.5%)	5 (1.8%)	0.65
Cirrhosis	0 (0%)	0 (0%)	0 (0%)	N/A
Malignancy	6 (4.4%)	1 (0.7%)	7 (2.6%)	0.06
Dementia	5 (3.7%)	4 (2.9%)	9 (3.3%)	0.73
Obesity (BMI >30.0)	50 (38.8%)	60 (44.8%)	110 (40.4%)	0.32
Median BMI (IQR)	29.0 (25.3 - 32.3), 129	28.9 (25.8 - 35.2), 134	29.0 (25.7 - 33.3), 263	0.12
No Listed Comorbidities[5]	27 (19.9%)	24 (17.6%)	51 (18.8%)	0.64
Pregnancy	2 (1.5%)	1 (0.7%)	3 (1.1%)	0.56
Homelessness	8 (5.9%)	8 (5.2%)	16 (5.9%)	0.79
**Symptoms**				
Fever	115 (84.6%)	110 (80.9%)	225 (82.7%)	0.42
Cough	110 (80.9%)	108 (79.4%)	218 (80.1%)	0.76
Dyspnea	107 (78.7%)	103 (75.7%)	210 (77.2%)	0.56
Chest Pain	25 (18.4%)	22 (16.2%)	47 (17.3%)	0.63
Diarrhea	29 (21.3%)	35 (25.7%)	64 (23.5%)	0.39
Myalgias	46 (33.8%)	42 (30.1%)	88 (32.4%)	0.60
Anosmia	9 (6.6%)	5 (3.7%)	14 (5.1%)	0.27
Altered Mental Status	8 (5.2%)	13 (9.6%)	21 (7.7%)	0.16
Headache	32 (23.5%)	20 (14.7%)	52 (19.1%)	0.06

Syncope	0 (0%)	3 (2.2%)	3 (1.1%)	0.08
Symptom Duration (Median, IQR)[6]	7 (4-8), 129	6 (3-8), 129	7 (4-8), 258	0.94
**NEWS Score (334)**				
Low Risk (0-4)	41 (30.2%)	27 (19.9%)	123 (36.5%)	<0.01
Medium Risk (5-6)	39 (28.7%)	26 (19.1%)	69 (20.5%)	-
High Risk (7+)	56 (41.2%)	83 (61.0%)	145 (43.0%)	-
NEWS (median, IQR)	6 (4-8), 135	7 (5-9), 134	6 (3-8), 334	<0.01
**Admission Laboratory Tests[7]**				
WBC (10^3/µl)	6.5 (5.2 - 9.4)	8.0 (6.0 - 11.0)	7.5 (5.6 - 10.4)	0.02
ANC (10^3/µl)	5.3 (3.7 - 7.8), 135	6.5 (4.7 - 9.4)	6.0 (4.3 - 9.1), 271	<0.01
ALC (10^3/µl)	1.0 (0.70 - 1.2), 135	0.8 (0.59 - 1.1)	0.9 (0.6 - 1.2), 271	0.20
AST >40 (U/L)	84 (63.2%), 133	106 (79.1%), 134	190 (70.9%), 268	<0.01
ALT >36 (U/L)	73 (54.9%), 133	81 (60.5%), 134	154 (57.5%), 268	0.36
CRP (mg/L)	109.8 (29.4 - 182.6), 119	171.9 (99.4 - 225.9), 113	147.0 (70.8 - 209.3), 232	<0.01
D-dimer (ng/mL)	261 (192 - 662), 106	430 (311 - 806), 107	378 (223 - 703), 213	0.30
Ferritin (ng/mL)	726 (348 - 1259), 93	972 (575 - 1494), 95	867.3 (470.8 - 1330.4), 188	0.03
LDH (IU/mL)	428 (321 - 552), 112	584 (459 - 774), 112	504.5 (378.0 - 674.5), 224	<0.01
Lactate >2.0 (mmol/L)	32 (35.2%), 91	33 (32.0%), 103	65 (33.5%), 194	0.65
Troponin >= 0.05 (ng/mL)	11 (13.3%), 83	32 (29.9%), 107	43 (22.6%), 190	<0.01

1: Means compared by two-sample t-test for continuous variables. X2 analysis performed for categorical variables.

2: Hypertension, Heart Failure, Stroke or Transient Ischemic Attack, Coronary Artery Disease

3: Asthma, Chronic Obstructive Pulmonary Disease (COPD), Obstructive Sleep Apnea, Interstitial Lung Disease

4: Chronic Kidney Disease (CKD) or End-stage Renal Disease (ESRD)

5: Patients lacking any comorbid conditions included in this table are included here.

6: Patients were omitted if diagnosis was >7 days after hospital admission.

7: Median with interquartile range reported for continuous variables; Proportion reported for categorical or dichotomous variables. # of patients with lab value available listed if missing values exist.

Comorbid Medical Conditions:

The majority of patients had at least one comorbid medical condition, with cardiovascular conditions (51.5%), obesity (40.4%), and type 2 diabetes (33.1%) being most prevalent (Table 1). Few patients had baseline pulmonary (13.2%) or renal (11.8%) disease. A total of 51 patients (18.8%) had none of these specifically assessed comorbidities. In general, presence of comorbid conditions did not differ by ICU admission except for chronic kidney disease (CKD), which was more prevalent in the ICU population (11.0% vs 2.9%, p<0.01). Only 14 patients (5.1%) reported being active smokers (Appendix Table 2).

Clinical Presentation:

The most common presenting symptoms were fever (82.7%), cough (80.1%) and dyspnea (77.2%). There were no significant differences when stratified by ICU admission, although altered mental

status was more common in patients requiring ICU admission (9.6% vs 5.2%, p = 0.16) and headache was more common in patients not admitted to the ICU (23.5% vs 14.7%, p = 0.06). Median duration of symptoms prior to presentation was similar in hypoxic patients managed on the floor (7 days) and in the ICU (6 days). Mean NEWS score was higher for patients admitted to the ICU (7.0 vs 5.9, p<0.01), and high-risk NEWS scores (7 or greater) were more prevalent in patients requiring ICU admission (61.0% vs 41.2%, p <0.01) (Table 1). Assessment of possible COVID-19 exposures and admission vital signs are included in Appendix Table 2.

Median white blood cell count (8.0 vs 6.5), neutrophil count (6.5 vs 5.3), CRP (171.9 vs 109.8), ferritin (972 vs 726), and lactate dehydrogenase (LDH) (584 vs 428) were higher in patients admitted to the ICU compared to those not admitted to the ICU; all mean values were also higher with a p value <0.05 via t-test (Table 1). Higher rates of troponin elevation (29.9% vs 13.3%) and AST elevation (79.1% vs 63.2%) were also seen in patients admitted to the ICU.

Treatment:

The majority of patients received at least one medication targeting SARS-CoV-2 virus (Table 2). The majority of patients received hydroxychloroquine (82.4%), with a higher rate of treatment in patients admitted to the ICU (90.5% vs 74.3%). The majority of patients admitted to the ICU received steroids (72.1%) compared to a minority of patients not admitted to the ICU (2.9%). Most patients also received antibiotics, with a higher rate of use in patients admitted to the ICU (80.9% vs 52.9%).

Table 2: Treatments, Respiratory Support, and Renal Injury

	Supplemental Oxygen, Never ICU (136)	Supplemental Oxygen, ICU (136)	Total (272)
**Treatment**			
Lopinavir/Ritonavir	15 (11.0%)	13 (9.6%)	28 (10.3%)
Hydroxychloroquine (HQ)	25 (18.4%)	10 (7.4%)	35 (12.9%)
HCQ + Azithromycin	76 (55.9%)	113 (83.1%)	189 (69.5%)
Any HCQ	101 (74.3%)	123 (90.5%)	224 (82.4%)
Tocilizumab	3 (2.2%)	27 (19.9%)	30 (11.0%)
Remdesivir Study Enrollment[1]	0 (0%)	4 (2.9%)	4 (1.5%)
Antimicrobials	72 (52.9%)	110 (80.9%)	182 (66.9%)
Steroids	4 (2.9%)	90 (72.1%)	94 (34.6%)
None of the Above	6 (4.4%)	2 (1.5%)	8 (2.9%)
**Highest Level of Respiratory Support**			
Oxygen - Nasal Cannula	91 (66.9%)	-	91 (33.4%)
Oxygen - Non-Rebreather	42 (30.9%)	5 (3.7%)	47 (17.3%)
NIV/HFNC	3 (2.2%)	9 (6.6%)	12 (4.4%)
Mechanical Ventilation	-	122 (89.7%)	122 (44.9%)
**Renal Injury**			
Baseline ESRD	8 (5.9%)	5 (3.7%)	13 (4.8%)
No ESRD	128 (94.1%)	131 (96.3%)	259 (95.2%)
No AKI	104 (81.3%)	31 (23.7%)	135 (52.1%)

AKI without RRT	23 (18.0%)	45 (34.4%)	68 (26.3%)
AKI with RRT	1 (0.8%)	55 (42.0%)	56 (21.6%)

[1]Receipt of remdesivir or placebo is unknown for these patients.
Abbreviations: NIV Non-invasive Ventilation: HFNC High Flow Nasal Cannula: AKI Acute Kidney Injury: RRT Renal Replacement Therapy: ESRD End-Stage Renal Disease

Patients managed outside the ICU mostly required nasal cannula alone (66.9%) but 45 of these patients (33.1%) required treatment with NRB or NIV/HFNC for severe hypoxia or respiratory distress. Nearly all patients admitted in the ICU required mechanical ventilation (89.7%) with only 14 (10.3%) requiring either NRB or NIV/HFNC as their highest level of respiratory support (Table 2).

Renal Injury:

Of patients requiring ICU admission, 100 of 131 patients without baseline ESRD (76.3%) developed AKI and 55 of these patients (42.0%) required RRT. Of hypoxic floor patients without ESRD (128), 23 had AKI without RRT (18.0%) and 1 patient required RRT (0.8%) (Table 2).

Hospital Outcomes:

Hospital outcomes stratified by pulmonary support and renal injury are available in Table 3. Mortality among those requiring supplemental oxygen was 28.7%: 52.9% (72 of 136) in the ICU and 4.4% (6 of 136) outside the ICU. Half of the patients with fatal outcome outside the ICU had AKI without RRT (3 of 6), and two-thirds required NRB or NIV/HFNC (4 of 6). The majority of deaths occurred in the ICU (72 of 78, 92.3%) and among patients requiring mechanical ventilation (67 of 78, 85.9%). The subset of patients requiring NRB or NIV/HFNC outside the ICU showed a trend towards higher mortality than those requiring nasal cannula alone (8.9% vs 2.2%, p = 0.074). Of ICU patients surviving to discharge or hospital day 30, 45 (33.1%) remained hospitalized with 22 (16.2% of total) still in the ICU; only 18 (13.2% of total) had been discharged home or to a care facility.

Table 3: Hospital Outcomes Stratified by Respiratory Support and Renal Injury

	**Totals**	**Mortality**	**Remain in ICU at HD 30**	**Remain on floor at HD 30**	**Discharge by HD 30**
**Totals**	**272**	**78 (28.7%)**	**22 (8.1%)**	**33 (12.1%)**	**137 (50.4%)**
**Supplemental Oxygen, ICU[1]**	**136**	**72 (52.9%)**	**22 (16.2%)**	**23 (16.9%)**	**18 (13.2%)**
NIV or NRB	14	5 (35.7%)	0 (0%)	2 (14.3%)	7 (50.0%)
Mechanical Ventilation[2]	122	67 (54.9%)	21 (17.2%)	21 (17.2%)	12 (9.8%)
ESRD	5	4 (80.0%)	0 (0%)	0 (0%)	1 (20.0%)
No AKI[2]	31	9 (29.0%)	5 (16.1%)	5 (16.1%)	12 (38.7%)
AKI without RRT[1,2]	45	23 (51.1%)[3]	7 (15.6%)	10 (22.2%)	4 (8.9%)
AKI with RRT[2]	55	36 (65.5%)[3]	10 (18.2%)	8 (14.5%)	1 (1.8%)
AKI with or without RRT[1,2]	100	59 (59.0%)	17 (17.0%)	18 (18.0%)	5 (5.0%)
**Supplemental Oxygen, Never ICU[1]**	**136**	**6 (4.4%)**	**-**	**10 (7.4%)**	**119 (87.5%)**
Nasal Cannula	91	2 (2.2%)	-	4 (4.4%)	85 (93.4%)

NIV or NRB/HFNC[1]	45	4 (8.9%)[4]	-	6 (13.3%)	34 (75.6%)
ESRD	8	0 (0%)	-	3 (37.5%)	5 (62.5%)
No AKI[1,2]	104	3 (2.9%)	-	4 (3.8%)	96 (92.3%)
AKI without RRT[2]	23	3 (13.0%)	-	2 (8.7%)	18 (78.2%)
AKI with RRT[2]	1	0 (0%)	-	1 (100%)	0 (0%)
AKI with or without RRT[2]	24	3 (12.5%)[5]	-	3 (12.5%)	18 (75.0%)

[1]Patients transferred to another hospital (2 in total) not included, % do not add to 100%

[2]Patients with baseline ESRD excluded from this analysis.

[3]Via X^2 analysis, using comparator group of no AKI, p=0.055 for AKI without RRT and p=0.001 for AKI with RRT

[4]Via X^2 analysis, using Nasal Cannula as comparator group, p = 0.074

[5]Via X^2 analysis, using No AKI as comparator group, p = 0.045

Abbreviations: HD Hospital Day: AKI Acute Kidney Injury: RRT Renal Replacement Therapy: NIV Non-invasive Ventilation: NRB Non-rebreather: HFNC High Flow Nasal Cannula: ESRD End-Stage Renal Disease

In critically ill patients without ESRD, AKI was associated with mortality without RRT (51.1% vs 29.0%, p = 0.055) and with RRT (65.5% vs 29.0%, p = 0.001). Among patients outside the ICU without ESRD, AKI was associated with mortality (12.5% vs 2.9%, p = 0.045).

In multivariate analysis of risk factors for mortality, controlling for demographics and baseline comorbid conditions, the strongest risk factors for mortality included dementia (aRR 2.11 95%CI 1.50-2.97), age 65 or older (aRR 2.0, 95%CI 1.33-3.0), obesity (aRR 1.36, 95%CI 1.07-1.74), and male sex (aRR 1.32, 95%CI 1.03-1.68). The effect of cardiovascular (aRR 1.12, 95%CI 0.74-1.68) and renal comorbidities (aRR 1.28, 95%CI 0.92-1.81) showed a trend towards higher mortality but did not reach statistical significance. Pulmonary conditions and type 2 diabetes were not associated with mortality (Table 4).

Table 4: Unadjusted and Adjusted Risk Ratios (RR) and 95% Confidence Intervals (CI) for Associations between Baseline Socio-demographic and Baseline Comorbidity[1] and COVID-19 Mortality (N=272 Patients Treated with Supplemental Oxygen)

Baseline Characteristics	N (%) Mortality	RR (95% CI)	Adjusted RR[2] (95% CI)
Age			
18-64 years (N=187)	41 (21.9)	Ref	Ref
65 years and older (N=85)	37 (43.5)	1.99 (1.38-2.85)	2.00 (1.33-3.00)
Sex			
Female (N=88)	22 (25.0)	Ref	Ref
Male (N=184)	56 (30.4)	1.22 (0.80-1.86)	1.32 (1.03-1.68)
Race			
White (N=25)	7 (28.0)	Ref	Ref
Black/Latinx (N=80)	24 (30.0)	1.07 (0.53-2.18)	0.98 (0.50-1.93)
Other, Asian, Native American (N=148)	43 (29.0)	1.04 (0.53-2.04)	1.04 (0.64-2.47)
Not Defined (N=19)	4 (21.1)	0.75 (0.26-2.20)	0.75 (0.45-3.22)
Cardiovascular Comorbidity[3]			
No (N=132)	32 (24.2)	Ref	Ref
Yes (N=140)	46 (39.9)	1.36 (0.92-1.99)	1.12 (0.74-1.68)
Pulmonary Comorbidity[4]			
No (N=236)	68 (28.8)	Ref	Ref
Yes (N=36)	10 (27.8)	0.96 (0.55-2.69)	0.87 (0.52-1.47)

Renal Comorbidity[5]			
No (N=240)	67 (27.9)	Ref	Ref
Yes (N=32)	11 (34.4)	1.23 (0.73-2.07)	1.28 (0.92-1.81)
Type 2 Diabetes			
No (N=182)	52 (28.6)	Ref	Ref
Yes (N=90)	26 (28.9)	1.01 (0.68-1.50)	0.85 (0.61-1.20)
Immunosuppression			
No (N=261)	76 (29.1)	Ref	Ref
Yes (N=11)	2 (18.2)	0.62 (0.18-2.22)	0.63 (0.17-2.36)
HIV			
No (N=267)	76 (28.5)	Ref	Ref
Yes (N=5)	2 (40.0)	1.41 (0.47-4.18)	1.48 (0.51-4.34)
Malignancy			
No (N=265)	77 (29.1)	Ref	Ref
Yes (N=7)	1 (14.3)	0.49 (0.08-3.05)	0.57 (0.09-3.58)
Dementia			
No (N=263)	71 (27.0)	Ref	Ref
Yes (N=9)	7 (77.8)	2.88 (1.93-4.31)	2.11 (1.50-2.97)
Obesity (Body Mass Index ≥30)			
No (N=153)	41 (26.8)	Ref	Ref
Yes (N=110)	35 (31.8)	1.19 (0.81-1.73)	1.36 (1.07-1.74)

[1]A total of 212 of 272 patients had at least one comorbidity upon hospitalization including cardiovascular, pulmonary, or renal comorbidity, diabetes, immunosuppression, HIV, malignancy, dementia, obesity (Body Mass Index ≥30). Presentation with any comorbidity was associated with 1.59 times the mortality risk (95%CI:0.88-2.87).

[2]All socio-demographic and comorbidity factors were included in the fully-adjusted model.

[3]Hypertension, Heart Failure, Stroke or Transient Ischemic Attack, Coronary Artery Disease

[4]Asthma, Chronic Obstructive Pulmonary Disease (COPD), Obstructive Sleep Apnea, Interstitial Lung Disease

[5]Chronic Kidney Disease (CKD) or End-stage Renal Disease (ESRD)

Of laboratory values assessed for association with mortality in adjusted analysis, elevated ferritin > 1000 mg/dL (RR 1.64 95%CI 1.04-2.61), LDH > 500 IU/mL (RR 2.13 95%CI 1.33-3.43), and troponin > 0.05 ng/mL (RR 1.58 95%CI 1.03 – 2.42) were associated with mortality. In subgroup analysis, the association of troponin with mortality was retained only for male patients and for those without known comorbid conditions, and for ferritin this association was retained only for female patients (Table 5).

Table 5: Unadjusted and Adjusted Risk Ratios (RR) and 95% Confidence Intervals (CI) for Associations between Selected Laboratory Indicators and COVID-19 Mortality (N=272 Patients Treated with Supplemental Oxygen)

Laboratory Indicators	N (%) Mortality	RR (95% CI)	Adjusted RR[1] (95% CI)
White Blood Cell >10.8 $10^3/\mu L$			
No (N=210)	59 (28.1)	Ref	Ref
Yes (N=62)	19 (30.7)	1.09 (0.71 -1.68)[2]<br>*<u>18-64 yrs</u>: 1.57 (0.89-2.78)*<br>*<u>65+ yrs</u>: 0.63 (0.32-1.23)*	1.08 (0.71 -1.64)[2]<br>*<u>18-64 yrs</u>: 1.74 (0.99-3.06)*<br>*<u>65+ yrs</u>: 0.70 (0.36-1.35)*
Absolute Neutrophil Count (ANC) >7.6 $10^3/\mu L$			
No (N=181)	47 (26.0)	Ref	Ref
Yes (N=90)	31 (34.4)	1.32 (0.91 -1.93)	1.28 (0.89-1.86)
Absolute Lymphocyte Count (ALC) <0.8 $10^3/\mu L$			
No (N=158)	21 (26.0)	Ref	Ref
Yes (N=113)	43 (32.7)	1.26 (0.87-1.83)	1.09 (0.75 -1.59)
Aspartate Transaminase (AST) >40 U/L			
No (N=80)	16 (20.0)	Ref	Ref

Yes (N=187)	60 (32.1)	1.60 (0.99-2.61)	1.83 (1.10-3.03)[2]<br>*18-64 yrs: 1.26 (0.66-2.40)*<br>*65+ yrs: 2.72 (1.19-6.28)*
C-reactive protein (CRP) >200 mg/L<br>No (N=169)<br>Yes (N=63)	 45 (26.6)<br>19 (30.2)	 Ref<br>1.13 (0.72-1.78)[2]<br>*18-64 yrs: 1.77 (0.98-3.19)*<br>*65+ yrs: 0.62 (0.28-1.37)*	 Ref<br>1.03 (0.66-1.59)[2]<br>*18-64 yrs: 1.82 (1.00-3.31)*<br>*65+ yrs: 0.57 (0.26-1.24)*
Ferritin >1000 ng/mL<br>No (N=107)<br>Yes (N=81)	 24 (22.4)<br>27 (33.3)	 Ref<br>1.49 (0.93-2.37)[3]<br>*Female: 2.47 (1.06 -5.78)*<br>*Male: 1.15 (0.66-2.00)*	 Ref<br>1.64 (1.04 -2.61)[3]<br>*Female: 3.59 (1.89-6.82)*<br>*Male: 1.30 (0.76-2.21)*
Lactate dehydrogenase (LDH) >500 IU/mL<br>No (N=107)<br>Yes (N=117)	 19 (17.8)<br>44 (37.6)	 Ref<br>2.12 (1.32 -3.39)	 Ref<br>2.13 (1.33-3.43)
Troponin >0.05 ng/mL<br>No (N=147)<br>Yes (N=43)	 39 (26.5)<br>19 (44.2)	 Ref<br>1.67 (1.08 -2.56)[3,4]<br>*Female: 0.92 (0.35-2.40)*<br>*Male: 2.10 (1.30-3.39)*<br><br>*No Comorbidity: 5.41 (2.64-11.09)*<br>*≥1 Comorbidity: 1.43 (0.89 -2.27)*	 Ref<br>1.58 (1.03-2.42)[3,4]<br>*Female: 0.81 (0.31-2.14)*<br>*Male: 1.97 (1.24-3.12)*<br><br>*No Comorbidity: 4.23 (1.93-9.27)*<br>*≥1 Comorbidity: 1.44 (0.92 -2.27)*

[1]Adjusted for age (65 years or older versus 18-64 years), sex, race, and presence of at least one comorbidity upon hospitalization including cardiovascular, pulmonary, or renal comorbidity, diabetes, immunosuppression, HIV, malignancy, dementia, obesity (Body Mass Index ≥30). Presentation with any comorbidity was associated with 1.59 times the mortality risk (95%CI:0.88-2.87).
[2]Age differences in association observed (p<0.15 for the laboratory indicator by age interaction term).
[3]Gender differences in association observed (p<0.15 for the laboratory indicator by gender interaction term).
[4]Differences in association by comorbidity status observed (p<0.15 for the laboratory indicator by comorbidity interaction term).

Discussion:

We describe clinical presentation and outcomes of patients admitted with COVID-19 to a large, safety-net hospital in NYC. In this exploratory analysis, we assessed differential characteristics and outcomes by severity of illness primarily by location of care (ICU vs non-ICU), with a focus on patients requiring supplemental oxygen. Our main findings include identification of dementia as well as obesity, age, and male sex as strong mortality risk factors, a finding of low mortality among patients with severe illness managed outside the ICU, and the clinical impact of renal injury for patients both in and outside the ICU.

Mortality among patients requiring supplemental oxygen, excluding those with asymptomatic carriage or mild disease not requiring supplemental oxygen therapy, was 29.8%, with the majority of deaths occurring in the ICU and among those receiving mechanical ventilation. Mortality rates in the ICU and with mechanical ventilation vary widely in previous reports (14.6%-88.1%). [4,10,12,16] The 30-day mortality rate in the BHC ICU (52.9%) was higher than some other NYC reports. This may have been influenced by BHC's status as a referral center for critically ill patients, including those requiring subspecialty care, for the NYCH+H system. Another potentially mediating factor is duration of symptoms prior to presentation. The median duration of symptoms in our population was longer (7 days) than

other NYC cohorts, although most studies do not report this variable and it is prone to recall and reporter bias. [10] One study from Seattle which showed similar mortality rate (50%) in critically ill patients had similar duration of symptoms prior to presentation. [9]

Mortality for those managed outside the ICU was 4.4%, including for those managed for severe hypoxia with escalation to NRB or NIV/HFNC, similar or lower than what was observed in other NYC cohorts. [4,12,16] Several interventions allowed for safe management of higher acuity patients outside the ICU, including the following: 1) An increase in continuous oxygen monitoring capabilities, allowing for rapid detection of decompensations and situations that required escalations in care; 2) Expansion of rapid response and code teams ensuring a timely response to these decompensating patients; 3) An increase in nurse staffing ratios on the floor to 4:1 from the standard of 6:1; and 4) Newly developed oxygenation protocols to guide frontline providers in management of these patients.

Risk factors associated with mortality reported in prior studies include advanced age, male sex, obesity, and other comorbidities, specifically cardiovascular conditions such as hypertension. [4,12,17,18] In our population, advanced age, male sex, and obesity were the main factors associated with mortality. Cardiovascular and renal comorbidities were not associated with mortality in an adjusted analysis, perhaps owing to a limited sample size and inability to detect these associations. Alternatively, our findings could suggest the relative importance of age and obesity in a younger population with fewer medical comorbidities. In contrast with other cohorts from NYC, where fewer than 10% of patients had no comorbid conditions, approximately one in five patients in our cohort had no recognized comorbid conditions. However, we cannot assess in our analysis whether the lower comorbidity rate is related to unrecognized comorbid conditions in a vulnerable population without adequate access to primary care services.

Low rates of baseline immunosuppression, HIV, and liver disease precluded adequate assessment of these comorbid conditions. Despite a small population of patients with pre-existing dementia, this comorbidity was strongly associated with mortality. This could reflect prior observations of increased mortality related to pneumonia among those with pre-existing dementia, or may be related to a lower likelihood of escalation of care if pre-existing do-not-resuscitate orders were in place. [19]

Laboratory abnormalities associated with mortality included ferritin and LDH. Additionally, markers of multi-organ involvement such as transaminase and troponin elevation were associated with mortality. Despite a relatively small cohort, subgroup analyses demonstrated interesting associations, including the correlation of ferritin with mortality specifically among female patients and the association of troponin elevation with mortality among male patients. These observations could help tailor laboratory assessment at the time of admission for patients diagnosed with COVID-19, and direct the development of predictive models in larger datasets in the future.

Despite a low prevalence of baseline renal comorbidities, AKI and need for RRT were seen in a high proportion of critically ill patients, consistent with other NYC reports. [4,10] The expanded need for RRT was unexpected, and has led to a significant strain on dialysis capability across hospital systems. [20,21] The need for expanded dialysis access should be expected for hospitals responding to a surge in COVID-19 illness unless current and future therapies employed against COVID-19 can mitigate the rate of renal injury in COVID-19 critical illness.

Rates of RRT in critically ill populations in NYC are similar across different hospitals, ranging from approximately 30-40% in most reports. [4,10,12] Many hypotheses for high rates of AKI and RRT in critical illness in COVID-19 have been proposed. As suggested by other researchers, the fluid-restrictive strategy in management of ARDS could cause unintended harm by worsening AKI, which may explain similar rates of RRT in ICU cohorts. [12] Additionally, pathologic studies have identified that viral entry and replication in renal cells via ACE-2 receptors may point to a direct impact on renal injury, which could be exaggerated during uncontrolled viral replication in critical illness. [22,23]

Strengths of this study include that data was gathered by manual chart review. This assures that information not well recorded in extractable areas in the EMR (e.g. symptoms and symptom duration) were accurately gathered. Among adult patients, only those incarcerated were excluded as a vulnerable population; therefore, this study is representative of the outbreak at our single institution for the first month of the COVID-19 outbreak in NYC.

This study has several limitations. As a retrospective chart review, data is restricted to what is documented in the EMR, with the possibility of patient-recall error or documentation error. The retrospective analysis is also restricted to the clinical data gathered, leading to missing data points. Detailed race and ethnicity were not recorded for many patients in this study, owing likely to limitations with demographic data entry during surge in clinical volume, limiting our ability to robustly analyze presentation and outcomes by race and ethnicity.

Conclusion

We describe a cohort of patients managed at a public hospital in NYC during the first month of the COVID-19 pandemic. BHC, along with the entire NYCH+H system, made significant structural changes to care for the vulnerable populations seen at our institution to provide a high level of care and mitigate to the extent possible the morbidity and mortality associated with COVID-19. Our results shed light on important clinical characteristics and outcomes in public hospitals. Future work is needed to continue to understand the pathophysiology of COVID-19 and develop best practices for the management of hospitalized patients with COVID-19.

References:

1. World Health Organization. Coronavirus disease 2019 (COVID-19) situation report-51. Geneva, Switzerland: World Health Organization; 2020. 2020.
2. New York City Department of Health, COVID-19: Data. <https://www1.nyc.gov/site/doh/covid/covid-19-data.page> Accessed July 21, 2020. 2020.
3. Preliminary estimate of excess mortality during the COVID-19 outbreak - New York City, March 11-May2, 2020. MMWR Morb Mortal Wkly Rep. 2020;69:603–5.
4. Richardson S, Hirsch JS, Narasimhan M, Crawford JM, McGinn T, Davidson KW, et al. Presenting characteristics, comorbidities, and outcomes among 5700 patients hospitalized with COVID-19 in the New York City area. JAMA - J Am Med Assoc. 2020;10022(20):E1–8.
5. Lewnard JA, Liu VX, Jackson ML, Schmidt MA, Jewell BL, Flores JP, et al. Incidence, clinical outcomes, and transmission dynamics of hospitalized 2019 coronavirus disease among 9,596,321 individuals residing in California and Washington, United States: a prospective cohort study. BMJ. 2020;369:m1923.
6. Petrilli CM, Jones SA, Yang J, Rajagopalan H, O'Donnell LF, Chernyak Y, et al. Factors associated

with hospitalization and critical illness among 4,103 patients with COVID-19 disease in New York City. BMJ. 2020;(369):m1966.
7. Zhou F, Yu T, Du R, Fan G, Liu Y, Liu Z et al. Clinical course and risk factors for mortality of adult inpatients with COVID-19 in Wuhan, China: A retrospective cohort study. Lancet. 2020;395(10229):1054–62.
8. Guan W, Ni Z, Hu Y, Liang W, Ou C, He J, et al. Clinical characteristics of coronavirus disease 2019 in China. N Engl J Med. 2020;382:1708-1720.
9. Bhatraju PK, Ghassemieh BJ, Nichols M, Kim R, Jerome KR, Nalla AK, et al. COVID-19 in critically ill patients in the Seattle region — Case series. N Engl J Med. 2020;382(21):2012–22.
10. Cummings MJ, Baldwin MR, Abrams D, Jacobson SD, Meyer BJ, Balough EM, et al. Epidemiology, clinical course, and outcomes of critically ill adults with COVID-19 in New York City: a prospective cohort study. Lancet. 2020;395(10239):1763-1770.
11. Grasselli G, Zangrillo A, Zanella A, Antonelli M, Cabrini L, Castelli A, et al. Baseline characteristics and outcomes of 1591 patients infected with SARS-CoV-2 admitted to ICUs of the Lombardy Region, Italy. JAMA. 2020;323(16):1574–81.
12. Argenziano MG, Bruce SL, Slater CL, Tiao JR, Baldwin MR, Barr RG, et al. Characterization and clinical course of 1000 patients with coronavirus disease 2019 in New York: retrospective case series. BMJ. 2020;369(March):m1996.
13. How New York City's public health care system responded to COVID-19. Wharton, University of Pennsylvania. May 21, 2020. Accessed June 28, 2020.
14. Harris PA, Taylor R, Thielke R, Payne J, Gonzalez N CJ. Research Electronic Data Capture (REDCap)-a metadata-driven methodology and workflow process for providing translation research informatics support. J Biomed Inf. 2009;42:377-381.
15. National early warning score (NEWS) - standardizing the assessment of acute-illness severity in the NHS. London, England. London, Engl R Coll Physicians. 2012;eISBN 978-1-86016-472-9.
16. Goyal P, Choi JJ, Pinheiro LC, Schenck EJ, Chen R, Jabri A, et al. Clinical characteristics of COVID-19 in New York City. [Letter to the Editor]. N Engl J Med. 382:2372–4.
17. Docherty AB, Harrison EM, Green CA, Hardwick HE, Pius R, Norman L, et al. Features of 20 133 UK patients in hospital with covid-19 using the ISARIC WHO Clinical Characterisation Protocol: Prospective observational cohort study. BMJ. 2020;369(March):1–12.
18. Gupta S, Hayek S, Wang W, Chan L, Mathews K, Melamed M et al. Factors associated with death in critically ill patients with coronavirus disease 2019 in the US. JAMA Int Med. 2020;
19. Foley N, Affoo R, Martin R. A systematic review and meta-analysis examining pneumonia-associated mortality in dementia. Dement Geriatr Cogn Disord. 2015;39:52–67.
20. Mahase E. COVID-19: increasing demand for dialysis sparks fears of supply shortage. BMJ. 2020;369:m1588.
21. Goldfarb DS, Benstein JA, Zhdanova O, Hammer E, Block CA, Caplin NJ, et al. Impending shortages of kidney replacement therapy for COVID-19 patients. CJASN. 2020;15(6):880–2.
22. Perico L, Benigni A RG. Should COVID-19 concern nephrologists? Why and to what extent? The emerging impasse of angiotensin blockade. Nephron Clin Pr. 2020;1–9.
23. Farkash EA, Wilson AC, Jentzen JM. Ultrastructural evidence for direct renal infection with SARS-CoV-2. JASN. 2020;31(7).

Supporting Information:

S1 Table: Data for patients not requiring supplemental oxygen

S2 Table: Smoking, exposures, presenting vital signs

Click here to access/download
Supporting Information
S1_Table.docx

Click here to access/download
Supporting Information
S2_Table.docx
