# Supplementary material for: Comorbidity and clinical factors associated with COVID-19 critical illness and mortality at a large public hospital in New York City in the early phase of the pandemic (March-April 2020)
Source: PLoS One. 2020 Nov 23;15(11):e0242760. doi: 10.1371/journal.pone.0242760 (PMC7682848; doi:10.1371/journal.pone.0242760)
Supplement: Attachment — Submitted filename: comments_08172020.docx [file pone.0242760.s003.docx]

**Review for PLoS ONE**

**Aug 17, 2020**

**Title: Comorbidity and Clinical Factors Associated with COVID-19 Critical Illness and Mortality at a Large Public Hospital in New York City in the Early Phase of the Pandemic (March-April 2020)**

This paper presents a study to describe patients admitted to NYC Health + Hospitals / Bellevue Hospital Center, in NYC, with COVID-19 in the month following the hospital’s first diagnosed case. Authors focused on comorbidities, clinical characteristics, and outcomes for patients admitted to ICU and non-ICU settings. However, there are questions that limit my enthusiasm of the paper, as outlined below.

1. Data Analysis:
   a. Authors considered IQR, median, frequency, t-test and chi-squared tests, while they weren’t introduced at data analysis section. Please explain the descriptive statistics (for both continuous and categorical variables) along with the association tests that were applied across manuscript including t-test and chi-squared test. (Numbers 1 and 7 of caption of Table 1 need to be transferred to the data analysis with more details.)
   b. Why authors considered parametric t-test compared with non-parametric methods? Is it based on known or unknown variance? Please justify this part since for the parametric t-test, the normality distribution needs to be assessed before applying t-test.
   c. As an example, Table shows the association between race and requiring supplemental Oxygen. How authors consider the chi-squared test for such analysis with few observations or even zero values? Please adjust such analysis by applying an appropriate statistical analysis (such as Fisher’s exact test) and add to the data analysis section as well.
   d. What is the statistical significant threshold for the statistical analyses across manuscript? Please add to the manuscript.
   e. Statistically, the logistic regression model is fitted not estimated. But we can consider the logistic regression model to estimate the OR and/or RR. In addition, bivariate analyses or bivariable analyses? Please clarify this part at data analysis section.
2. Results:
   a. For NEWS score, authors considered mean or median? Page 13, authors mentioned mean, so please be consistent across manuscript to report descriptive statistics.
   b. Table 1, not easy to follow. For example for symptom duration or NEWS, it’s supposed to have (median, IQR), but what are those numbers after the IQR values? If they represent the total observations then please modify the table and re-do the Table in better way.
   c. Table 1, for the CRP (admission lab test), please report all required values.
   d. Please define “other” group in race as a caption or across the manuscript.
   e. Why authors report cirrhosis (medical history), while it doesn’t show any observation/data?
   f. Table 3: not easy to follow the association analysis for outcomes. Please add the P values (in the caption) to the Table and make it easier to follow. In addition, apply the previous comments regarding the chi-square test.
   g. For the sum of the percentage of contingency table, it should be always 100%. So please modify the Table and exclude those two patients. In addition, the sum of the supplemental Oxygen, ICU is 135 not 136? Please modify Table 3 not easy to follow.
   h. For the adjusted logistic model, how the authors decide which variables or covariates need to be included in the model to adjust the fitted model?
