# Supplementary material for: Comorbidity and clinical factors associated with COVID-19 critical illness and mortality at a large public hospital in New York City in the early phase of the pandemic (March-April 2020)
Source: PLoS One. 2020 Nov 23;15(11):e0242760. doi: 10.1371/journal.pone.0242760 (PMC7682848; doi:10.1371/journal.pone.0242760)
Supplement: Attachment — Submitted filename: Response to Reviewers.docx [file pone.0242760.s005.docx]

**Reviewer #1:**

1. Table 1: means for skewed continuous variables (labs) need to be compared using a non-parametric test, such as the Kruskal-Wallis test, not a between-group t-test, which assumes normal distributions.

Response: Thank you for noting this. We have now modified Table 1 to compare group differences on continuous variables using the non-parametric Wilcoxon rank-sum test. We have updated our methods section to reflect this: “Socio-demographics, clinical characteristics, and laboratory markers at admission were compared across patients with COVID-19 illness who did and did not require admission to the ICU. Given a non-normal distribution of continuous variables, as determined by a Shapiro-Wilk normality test, we presented the median and interquartile range for continuous variables and compared continuous variables using Wilcoxon rank-sum tests. For categorical variables, we reported count and proportion for each group, and used Fisher’s exact test to compare distributions across categorical variables due to the small size of the sample.” (Page 4, Lines 17-23).

We have also updated the footnote in table 1: “All variables were calculated for the full sample of 270 patients, unless different (n) indicated in variable column. Median and interquartile range (IQR) presented for continuous variables (age, BMI, symptom duration, NEWS score, and some lab results). Count and proportion presented for remaining categorical variables.” (Page 7) and have updated p-values accordingly in Table 1.

2. The authors state “... altered mental status was more common in patients requiring ICU admission (9.6% vs 5.2%, p = 0.16) ..“ When a difference is not significant, the proper way to report it would be something like “ ...altered mental status did not differ significantly between those who did vs did not require ICU admission (9.6% vs 5.2%, p = 0.16). When the p value is > .05 and < 0.10, it may be acceptable to report a difference ‘with a trend level of significance’.

Response: We agree that this statement did not reflect statistical significance as defined in our paper. Therefore, we have redacted this statement and updated this sentence to now read: “There were no significant differences in symptom presentation when stratified by ICU admission.” (Page 7, Lines 11-12)

3. It is confusing to have “Supplemental Oxygen” in the table headings in Tables 1 and 2. I would suggesting removing this so it is clear the columns refer to ICU and no-ICU.

Response: We agree that this is confusing for the reader. We have updated each table to specify that the population under study is all patients requiring supplemental oxygen (e.g. “Table 1: Sociodemographics, Medical History, and Admission Laboratory Markers for Patients with COVID-19 Illness Requiring Supplemental Oxygen”, Page 5). The headings in tables 1, 2, and 3, and Supporting Table 2 now read “Never ICU” and “ICU”.

4. * Interactions are mentioned in the Method section and are shown in Table 5, but are never discussed in the Results or Discussion.

Response: Interactions are listed in the methods section and in Table 5 and specified when considered in the footnote of Table 5. We have added to the results section to discuss these associations. These paragraphs now read: “Of laboratory values assessed for association with mortality in adjusted analysis, elevated ferritin (>1000 mg/dL;aRR 1.66 95%CI 1.05-2.63), lactate dehydrogenase (LDH >500 IU/mL; aRR 2.12 95%CI 1.32-3.41), elevated aspartate transaminase (AST >40 U/L; aRR 1.84, 95% CI 1.11 – 3.04) and troponin (>0.05 ng/mL;aRR 1.57 95%CI 1.03 – 2.41) were associated with mortality in the overall sample. Neutrophil and lymphocyte counts were not associated with mortality in adjusted or unadjusted analysis. (Table 5). (Page 11, Lines 10-15)

“In subgroup analysis, we observed substantial variation in the association between laboratory values and mortality by age, sex, and comorbidity status. Elevated white blood cell counts and c-reactive protein levels were risk factors for mortality in those aged 18-64 years (WBC >10.8 10^3^/μL; aRR 1.75, 95% CI 1.00-3.08: CRP >200 mg/L; aRR 1.83, 95% CI 1.01-3.31) but were not associated with mortality among those 65 years or older (WBC aRR 0.70, 95% CI 0.36-1.35: CRP aRR 0.57, 95% CI 0.26-1.24) . Elevated ferritin was a mortality risk factor for females (>1000 mg/dL; aRR 3.60, 95% CI 1.90-6.83) but not males (aRR 1.32, 95% CI 0.77-2.24). Elevated troponin was a risk factor for mortality for males (>0.05 ng/mL; aRR 1.95, 95% CI 1.23 – 3.09) but not females (aRR 0.81, 95% CI 0.31 – 2.15). Elevated troponin was also a risk factor among those with no comorbid conditions (aRR 4.25, 95% CI 1.94 – 9.31) while it was weakly and insignificantly associated with mortality among those with at least one comorbidity (aRR 1.43, 95% CI 0.91-2.25).” (Page 12, Lines 8-18)

**Reviewer #2:**

Persons with dementia seem more sensitive to certain types of immune challenge and show higher mortality. Are you able to do any further analysis of the dementia group to suggest reasons for their susceptibility to death from Covid-19?

Response: We appreciate this observation. We acknowledge that due to our limited sample size, and small group of patients with dementia (9 patients) that any significant observations about this group will be limited. We reviewed these patients specifically and found that many had pre-existing DNR/DNI orders at the time of their death, but this data was not reviewed for our full population, limiting analysis. We have also noted that this population was older than the general population of those aged 65 or greater, meaning that there was residual confounding by age which we did not account for in our age adjustment in models.

We have added the following to the manuscript: “On dedicated review, we noted that do-not-resuscitate orders were in place for 5 of the 7 patients with dementia at the time of death, and therefore a lower likelihood of care escalation with measures such as resuscitation or intubation may have led to higher rates of mortality. However, code status was unavailable for the full cohort, limiting comparative analyses. Additionally, all patients with dementia were over the age of 65, and compared to the full cohort of individuals over 65, their age was on average 8 years greater. This raises the possibility of additional confounding based on more advanced age not accounted for in our adjustment by age category.” (Page 13, Lines 35-42)

Given the rapid developments in Covid-19 treatment could the authors make additional comments on the treatment regimens or recent relevant studies.

Response: We appreciate this author’s observations. While the goal of our study was not to comment specifically on treatments or on efficacy, we do believe we add to the available literature on treatment strategies which were prevalent prior to the availability of literature describing efficacy. We have added to the manuscript: “Regarding treatment of patients with COVID-19 illness, this cohort of patients notably presented during a period when scant evidence was available for guidance of treatment strategies. We observed high rates of hydroxychloroquine usage, although subsequent observational and randomized trials have failed to show significant benefit in treatment of COVID-19 illness. [25-27] Steroids were employed frequently for critically ill patients in our study but rarely used for patients requiring supplemental oxygen outside the ICU. Steroid therapy has subsequently shown promise to reduce mortality in patients receiving mechanical ventilation and supplemental oxygen compared to usual care. [28] We anticipate that many of the patients requiring supplemental oxygen but not requiring ICU admission would be treated with steroids under current treatment guidelines. Fewer numbers of critically ill patients received tocilizumab, likely owing to limited familiarity with this medication and a higher perceived risk profile. The role of targeted immune suppressants such as tocilizumab remains to be elucidated; while early observational data suggested a benefit, subsequent randomized trials have not demonstrated benefit. [29] Remdesivir, which had limited availability during this study period through a randomized trial, has subsequently shown promise in reducing time to recovery although did not demonstrate a significant mortality reduction in a randomized trial. [30] Finally, we observed high rates of concurrent antibiotic usage for management of patients both in and outside the ICU despite low rates of documented co-infection. [31] Widespread usage of broad-spectrum antimicrobials without clear benefit outside of documented bacterial infection raise the concern about accelerating antimicrobial resistance in vulnerable populations in the face of the pandemic.” (Page 14, Lines 21-39). References 25-31 were added (Page 16, Lines 37-48 and Page 17, Lines 1-6).

**Reviewer #3:** 

Comments
.Abstracts
n= to be inserted for the number of subjects i.e n=127

Response: n=270 added (Page 2, Line 13)

.Methods
Data
For the ‘team of students’, who are the students to be clearly stated.

Response: All individuals who participated in data acquisition and interpretation are listed as authors on the paper. Therefore, we removed this statement to avoid confusion on the part of readers. This sentence now reads “Retrospective manual data extraction was conducted using a standardized data capture tool.” (Page 3, Line 23)

Data analysis
All statistical tests used in the results section to be clearly stated in statistical analyses section. More information to be provided after this statement ‘testing effect modification by sex, age and comorbidity at the 0.15 level’. The acceptance level of statistical significant to be stated.

Response: We have clarified the statistical threshold for significance in the methods section: “All statistical tests were considered to be significant at the p<0.05 level, with the exception of the interactions tests that were considered significant at the p<0.15 level; the significance level for interaction analyses was increased to avoid type II error. [16]” (Page 5, Lines 1-3)

Results
Table 1, n= to be employed to indicate total sample. X^2 to be replaced with the actual ‘chi square' symbol.
n(%), N(%) to be placed in the first row. Individual symbol % for individual figures to be omitted. Statistical tests to be clearly denoted in table footnote.\

Response: All tables were updated to improve the ability of readers to interpret the data reported. Column headings state either n(%) or median (IQR) as appropriate, and a footnote was added to each table to assist with reader comprehension. Individuals %s were retained in the table, which we believe assists the readability of the tables. Statistical tests now are run via Fischer’s exact test, and chi-square symbol was removed. Data presentation and statistical tests are now summarized in the footnotes of Table 1: “All variables were calculated for the full sample of 270 patients, unless different (n) indicated in variable column. Median and interquartile range (IQR) presented for continuous variables (age, BMI, symptom duration, NEWS score, and some lab results). Count and proportion presented for remaining categorical variables.

^1^p-values are presented for Wilcoxon rank-sum tests for continuous variables and Fisher’s exact tests for categorical variables.” (Page 6)

Table 1, under Admission Laboratory Tests, n to be placed after the variable name and not after median (IQR) values (Likewise with Supplemental Table 1). The categorical variables and the continuous variable to be clearly separated with space and indicated with their respective p value. Row alignment for CRP to be improved. Symbol >= to be replaced with symbol ≥ (likewise in Supplemental Table 1). In Supplemental Table 2 <= to be replaced with ≤)

Response: CRP row alignment fixed in Table 1 (Page 6). Symbols replaced. Categorical and continuous variables were separated in Table 1 and Supplemental Table 1 for improved readability.

Table 2 & 3 n(%) N(%) to be placed in the first row. Symbol % for individual figure to be omitted. Likewise with Supplemental Table 1 & Supplemental Table 2.

Response: Tables modified as above, but % symbol was retained as above.

Table 2, NRB to be placed after Oxygen-Non-Rebreather.

Response: “(NRB)” added (Page 8).


Treatment
The sentence ‘The majority of patients received hydroxychloroquine (82.4%)’ to be revised and match the variable name in Table 2. HQ and any HCQ to be clearly defined/denoted in table footnote.

Response: This sentence was revised to now read: “The majority of patients received at least one medication targeting SARS-CoV-2 virus (Table 2). The majority of patients received hydroxychloroquine (HCQ; 82.4%), with a higher rate of treatment in patients admitted to the ICU (90.4% vs 74.1%).” (Page 8, Lines 2-3)

Hospital outcomes
Table 3, footnote X^2 to be replaced with symbol ‘chi square' .

Response: Chi-square no longer used, and symbol was removed.

Table 3 to be cited after ‘ In critically ill patients …. P=0.045)

Response: Table 3 is cited as appropriate (Page 10, Lines 5-6).

Clinical Presentation
For ‘Mean NEWS score was higher for patients admitted to the ICU (7.0 vs 5.9, p<0.01)’, median was reported in Table 1.

Response: Median NEWS score cited in the manuscript to be consistent with the results reported in the table. This sentence now reads: “Median NEWS score was higher for patients admitted to the ICU (7 vs 6, p=0.002), and high-risk NEWS scores were more prevalent in patients requiring ICU admission (p =0.008) (Table 1).” (Page 7, Lines 13-15)

Table 4 & 5, small n to be used to indicate individual sample size. N for overall total.

Table 5, the description for superscript 2,3,4 in the footnote requires fine tuning.

Response: “n=” added in tables 4 and 5. Footnotes updated for Table 5 based on this feedback. Footnotes now read: “^1^Adjusted for age (65 years or older versus 18-64 years), sex, race, and presence of at least one comorbidity upon hospitalization (cardiovascular, pulmonary, or renal comorbidity, diabetes, immunosuppression, HIV, malignancy, dementia, obesity).

^2^Interactions tested for age, sex, and presence of any comorbidity. Stratified estimates only included for interactions that were significant at the p<0.15 level.” (Page 12)

Symbol colon : for the sub title to be omitted.

Response: The “:” was retained as it signifies our short title, as requested by the journal.

Some references were not conformed to the journal format.

Response: References 1, 2, 13, 15, and 19 were updated. Reference 16 was added as part of the methods section. (Pages 15-16) They now read:

1: World Health Organization. Coronavirus disease 2019 (COVID-19) situation report-51. Geneva, Switzerland: World Health Organization; 2020. [Cited 21 July, 2020] Available from: https://apps.who.int/iris/handle/10665/331475.

2: New York City Department of Health, COVID-19: Data. [Cited 21 July, 2020]. Available from: https://www1.nyc.gov/site/doh/covid/covid-19-data.page

13: How New York City’s public health care system responded to COVID-19. Wharton, University of Pennsylvania. 21 May, 2020. [Cited June 28, 2020] Available from: https://knowledge.wharton.upenn.edu/article/new-york-citys-public-health-care-system-responded-covid-19/.

15: National early warning score (NEWS) - standardizing the assessment of acute-illness severity in the NHS. Report of a working party. London: RCP, 2012.

19: Gupta S, Hayek S, Wang W, Chan L, Mathews K, Melamed M et al. Factors associated with death in critically ill patients with coronavirus disease 2019 in the US. JAMA Int Med. 2020 Jul 15;e203596.

References 16, and 25-31 are new (Page 16).

Reference formatting was also updated to include page #s if now available from journal prints (added for references 4 and 24). If journal pages were unavailable for articles available only online, doi numbers were added as per journal guidelines (References 5, 6, 11, 18, 19, 21).

Tables formatting to follow journal format.

Response: Journal guidelines were reviewed, and table formatting was carried out as outlined above and in other reviewer comments. We believe that the tables fit formatting guidance provided on the PLOS ONE author guidelines website.

**Reviewer #4:**

1. Data Analysis:
a. Authors considered IQR, median, frequency, t-test and chi-squared tests, while they weren’t introduced at data analysis section. Please explain the descriptive statistics (for both continuous and categorical variables) along with the association tests that were applied across manuscript including t-test and chi-squared test. (Numbers 1 and 7 of caption of Table 1 need to be transferred to the data analysis with more details.)

Response: Thank you for pointing out this confusion. We have now simplified table 1 to include only median and IQR for continuous variables and counts and proportions for categorical variables. We have also applied Fisher’s exact test rather than Pearson chi-squared tests throughout given many variables had small cell counts. We also used Wilcoxon rank sum tests rather than t-tests when continuous given most variables were not found to be normally distributed when tested for with a Shapiro Wilk test of normality. We have edited the table captions and methods section accordingly. The methods section now reads: “Socio-demographics, clinical characteristics, and laboratory markers at admission were compared across patients with COVID-19 illness who did and did not require admission to the ICU. Given a non-normal distribution of continuous variables, as determined by a Shapiro-Wilk normality test, we presented the median and interquartile range for continuous variables and compared continuous variables using Wilcoxon rank-sum tests. For categorical variables, we reported count and proportion for each group, and used Fisher’s exact test to compare distributions across categorical variables due to the small size of the sample.” (Page 4, Lines 17-23)

b. Why authors considered parametric t-test compared with non-parametric methods? Is it based on known or unknown variance? Please justify this part since for the parametric t-test, the normality distribution needs to be assessed before applying t-test.

Response: As noted above, we used Shapiro-Wilk test to assess normality of continuous variables, and given most variables were not normally distributed, we modified our analyses to Wilcoxon rank-sum non-parametric tests rather than t-tests for all continuous variables. See response at ‘a’ above.

c. As an example, Table shows the association between race and requiring supplemental Oxygen. How authors consider the chi-squared test for such analysis with few observations or even zero values? Please adjust such analysis by applying an appropriate statistical analysis (such as Fisher’s exact test) and add to the data analysis section as well.

Response: Thank you for this suggestion. Given the small sample and cell sizes, we modified our statistical analysis comparing variables to a Fisher’s exact test rather than a chi-squared test throughout tables 1-3.

d. What is the statistical significant threshold for the statistical analyses across manuscript? Please add to the manuscript.

Response: We have clarified the statistical threshold for significance in the methods section as follows: “All statistical tests were considered to be significant at the p<0.05 level, with the exception of the interactions tests that were considered significant at the p<0.15 level. The significance level for interaction analyses was increased to avoid type II error. [16].” (Page 5, Lines 1-3)

e. Statistically, the logistic regression model is fitted not estimated. But we can consider the logistic regression model to estimate the OR and/or RR. In addition, bivariate analyses or bivariable analyses? Please clarify this part at data analysis section.

Response: We have modified the text to properly describe our fitting of the regression model and estimating of the RR. We also modified our term to “bivariate” throughout. The pertinent portion of the methods section now reads: “Using binomial regression, we estimated unadjusted and adjusted risk ratios (RRs) and 95% conﬁdence intervals (CIs) for associations between socio-demographic and comorbidity factors and mortality due to COVID-19 among patients requiring supplemental oxygen. Multivariable models included adjustment for all socio-demographic and comorbidity factors in order to estimate independent associations between independent variables and mortality. We also estimated RRs and 95% CIs for associations between laboratory indicators and mortality. Because laboratory indicators may offer improved clinical utility for specific subgroups based on demographics and/or presence of preexisting conditions, we tested for effect modification of the association between each laboratory indicator and mortality by age, sex, and presence of any comorbidity (cardiovascular, pulmonary, or renal comorbidity, diabetes, immunosuppression, HIV, malignancy, dementia, and obesity). In cases where associations differed by background factors (age, sex, presence of comorbidity), as indicated by significance of the background factor by laboratory indication product interaction term (P, 0.15), subgroup associations were presented. Otherwise, associations were presented in the overall sample to preserve statistical power and included adjustment for age, sex, and presence of any comorbidity. All models estimating associations between laboratory factors and mortality were adjusted for race/ethnicity; however, we did not assess effect modification by race/ethnicity because low cell counts in some race categories would not have supported reliable estimation of associations.” (Page 4, Lines 24-40)

2. Results:
a. For NEWS score, authors considered mean or median? Page 13, authors mentioned mean, so please be consistent across manuscript to report descriptive statistics.

Response: We appreciate this suggestion. As above, we have modified the test to report only median NEWS score for consistency. This sentence now reads: “Median NEWS score was higher for patients admitted to the ICU (7 vs 6, p=0.002), and high-risk NEWS scores were more prevalent in patients requiring ICU admission (p =0.008) (Table 1).” (Page 7, Lines 13-15)

b. Table 1, not easy to follow. For example for symptom duration or NEWS, it’s supposed to have (median, IQR), but what are those numbers after the IQR values? If they represent the total observations then please modify the table and re-do the Table in better way.

Response: We have modified Table 1 to include the number of observations in the variable column and have clarified this in the footnotes of the table. We have applied this consistently in the variable columns in Table 1 and Supporting Table 1.

c. Table 1, for the CRP (admission lab test), please report all required values.

Response: Prior formatting limited the readability of the CRP lab test. This is now fixed and can be seen more clearly in Table 1. (Page 6)

d. Please define “other” group in race as a caption or across the manuscript.

Response: We have added information in the methods section defining race and ethnicity categories across the manuscript. This added methods section reads: “Race and ethnicity were gathered from patient-reported data entered into the EMR. Options available for race in the EMR included American Indian or Alaskan, Asian, Black, Native Hawaiian, Other Pacific Islander, White, Other, and Unknown/Not Recorded. Ethnicity included assessment of Hispanic / Latinx or Non-Hispanic identity. All patients who reported Hispanic / Latinx ethnicity reported “Other” or “Unknown/Not Recorded” race and were categorized as “Hispanic / Latinx” in a condensed assessment of race and ethnicity.” (Page 3, Lines 37-39 and Page 4, Lines 1-3)

e. Why authors report cirrhosis (medical history), while it doesn’t show any observation/data?

Response: We appreciate this observation. We have removed cirrhosis, which was only present in the cohort reported in Supporting Table 1. A footnote specifies that no patients in Table 1 had chronic liver disease or cirrhosis: “^6^Patients lacking all comorbid medical conditions included in this table. No patients in this sample had chronic liver disease or cirrhosis, which is omitted.” (Page 7), and the row for cirrhosis has been removed to avoid confusion.

f. Table 3: not easy to follow the association analysis for outcomes. Please add the P values (in the caption) to the Table and make it easier to follow. In addition, apply the previous comments regarding the chi-square test.

Response: We agree that this table was challenging to follow. We have added column headings to demonstrate the subgroups analyzed based on respiratory support and renal injury. P-values previously reported in footnotes are now reported in a separate column with a footnote denoting that p-values refer to mortality, with reference groups clearly delineated. (Page 9)

g. For the sum of the percentage of contingency table, it should be always 100%. So please modify the Table and exclude those two patients. In addition, the sum of the supplemental Oxygen, ICU is 135 not 136? Please modify Table 3 not easy to follow.

Response: We appreciate this reviewer’s comments. We reconsidered the population on which we carried out primary analyses and recognized that we could not assess 30-day outcomes for these two patients transferred outside our institution. We therefore have removed these 2 patients from the primary analysis and have added information at the beginning of the results section delineating our choice of the primary population for analysis. This introductory paragraph now reads: “Between March 9^th^ and April 8^th^, 2020, a total of 337 adult patients eligible for inclusion in this study were diagnosed with COVID-19 at BHC. This cohort was split first by need for supplemental oxygen, with 65 patients (19.3%) never requiring supplemental oxygen. This group represented a heterogeneous population, including many admitted from congregate settings early in the outbreak for social isolation and individuals already hospitalized for other reasons (e.g. inpatient psychiatric care, acute inpatient rehab). Details regarding the 65 patients never requiring supplemental oxygen are available in Supporting Table 1. Of the remaining 272 patients requiring supplemental oxygen, 2 patients were transferred to outside hospitals prior to hospital day 30, and therefore 30-day hospital outcomes were unable to be assessed. Primary analyses were conducted among the remaining patients who required supplemental oxygen (n=270).” (Page 5, Lines 8-17)

h. For the adjusted logistic model, how the authors decide which variables or covariates need to be included in the model to adjust the fitted model?

Response: Initial selection of variables to include in the bivariate regression model for risk of mortality (Table 4) was based on a priori knowledge of potential demographic and clinical risk factors for negative COVID-19 outcomes. These were taken from several references cited in our paper, notably: Petrilli et al, reference 6; Cummings et al, reference 10; Gresselli et al, reference 11; Argenziano et al, reference 12.

For adjusted models, we included all comorbidities as covariates given our interest in assessing independent associations of each covariate with mortality. We have now included this information more clearly in the methods section, as listed in response to reviewer 4, section 1e.

For tests of associations between admission lab test values and mortality, we conducted bivariate and multivariable analyses adjusted for age, sex, race, and presence of any comorbidity. We limited the adjusted analyses to these covariates given specific comorbidities may be correlated with certain lab test values, and adjusting for all comorbidities may have obscured associations between lab values and risk of death.

**Reviewer #5:** Thank you for the opportunity to review the well reported manuscript.

In general, the data analyses were appropriately performed and reported. Minor amendments with a recheck on percentage calculations throughout all tables were needed for a quality presentation. Detailed comments that focused mainly on the data analysis can be found from the attachment.

Response: We appreciated this reviewer’s comments. We rearranged Table 1 to clarify the confusion mentioned by this author, including comments included above. Fisher’s exact test was used to compare categorical variables throughout our manuscript given comparison of relatively small populations.
